# Allele and genotype frequencies of β-lactoglobulin gene using PCR-RFLP in Algerian local cattle populations

**DOI:** 10.22099/mbrc.2023.47661.1841

**Published:** 2024

**Authors:** Nadjet Boushaba, Nacera Tabet-Aoul

**Affiliations:** 1 Laboratory Genetics Molecular and Cellular, Department of Molecular Genetics Applied, Faculty of Nature and Life Sciences, University of Science and Technology of Oran "Mohamed Boudiaf ", B.P 1505 El M’Naouar, 31000 Oran, Algeria; 2 Department of Biotechnology, Faculty of Nature and Life Sciences, University of Oran 1 "Ahmed Ben Bella", 31000 Oran, Algeria

**Keywords:** Algerian cattle, β-lactoglobulin, Genetic variation, PCR-RFLP method, SNP

## Abstract

Milk protein genetic polymorphisms are associated with economically important traits in dairy cattle. The objective of this study is to genotype a single nucleotide polymorphism (SNP) responsible for the amino acid changes in the beta-lactoglobulin (β-Lg) variants A and B on 85 unrelated DNA representing Algerian cattle populations: Chelifienne (28), Cheurfa (31) and Guelmoise (26). The method used is the PCR-RFLP (Polymerase Chain Reaction-Restriction Fragment Length Polymorphism). Genetic polymorphism was detected by digestion of PCR products amplified of exon II of β-Lg gene by with the endonuclease *Hae*III enzyme. The results revealed that the amplified product was observed as 247 bp. Restriction digestion with *Hae*III revealed three genotypes: AA, AB and BB. The genotypic frequencies of AA, AB and BB genotypes were 0.08, 0.41, 0.50; 0.08, 0.41, 0.50 and 0.01, 0.19, 0.56 in Chelifienne, Cheurfa and Guelmoise and respectively. Frequency of AA genotype was absent in Guelmoise population. Frequencies of A and B alleles were 0.29 and 0.71 in both Chelifienne and Cheurfa and 0.25 and 0.75 Guelmoise population. These results further confirm that Bos torus cattle are predominantly of β-Lactoglobulin B type. The Chi-square test at *p*-value < 0.05 results revealed that the Chelifienne and Cheurfa populations were in Hardy-Weinberg equilibrium and the results are not significant for the Guelmoise. This genetic information could be useful to estimate the effect of polymorphism on different milk production of Algerian bovine populations.

## INTRODUCTION

The protein, β-lactoglobulin (β-Lg) constitutes a significant proportion ~10% of the total protein in cow's milk and is a major element of the whey protein fraction [1]. β-lactoglobulin is found in the milk of ruminant animals however is not present in the human, mice and rats milk,. These proteins are believed to play a role in transporting small hydrophobic substances. In particular, β-lactoglobulin has been associated with facilitating the absorption of vitamin A from milk [2] and potentially impacting enzyme activity [3]. Polymorphism analysis of lactoproteins within the *Bos* *genus* has been conducted across various cattle breeds, including *Bos taurus*. Others studies have examined polymorphism in populations of zebu (*Bos indicus*) and yaks (*Bos grunniens*) [4]. Polymorphisms in the β-Lg gene have been linked to increased concentrations of β-Lg protein [5]. As a result, milk protein genes hold potential as genetic markers for incorporating additional selection criteria in dairy cattle breeding programs [6]. 

The bovine β-Lg gene has been successfully sequenced, revealing its structure. It consists of seven exons in a total of 4724 bp [7] encompassing an ∼5 kb genomic segment [8] and has been mapped to position q28 on chromosome 11 of the cow [9] and occurs as a dimer, with a molecular weight of 18,000 per monomer. However, it is worth noting that β-lactoglobulin found in other animals such as pigs, horses and donkeys exists as a monomer [10]. In cattle, twelve polymorphic variants of β-Lg have been identified (A, B, C, D, E, F, G, W, H, I, J, and X). These variants correspond to different protein forms [11]. However, the two most common variants, A and B, have been specifically linked to variations in milk protein yield and composition [12].

The cDNA encoding for β-lactoglobulin (β-LG) sequenced in many ruminant species, including bovine [13], ovine [14], and caprine [15]. As already noted the coding sequence (486 bp coding for 162 amino acids) is highly conserved in the three species, the positions of amino acid substitutions have been identified for five of these variants, A, B, C, D, and E [16].

The β-Lg A and B variants differ by two amino acid substitutions in the polypeptide chain arising from two single-nucleotide substitutions in β*-Lg-I* gene: in the A variant, Aspartic acid 64 (GAT) is changed to Glycine (CGT), while Valine118 (GTC) is changed to Alanine (GCC) in the B variant [16]. The change at position 118 creates a *Hae*lll restriction site (GG/CC) in β-lactoglobulin B not present in β-lactoglobulin A [17].

Traditionally, the genetic typing of milk was carried out using electrophoretic separation of milk proteins on starch and polyacrylamide gels [18]. This method involved subjecting milk protein samples to electrophoresis, a process where an electric field is applied to separate proteins based on their charge and size. Starch and polyacrylamide gels were commonly used as the medium for protein separation during this traditional approach. By analyzing the resulting protein banding patterns on the gels, researchers were able to determine the genetic variants or polymorphisms present in milk proteins, including β-lactoglobulin and other milk protein elements.

Indeed, while protein electrophoresis has been a valuable tool for detecting genetic polymorphisms in milk proteins, it has limitations in terms of coverage and resolution. The level of observed polymorphism in proteins is often limited, and this technique analyzes only a small number of loci, providing a partial representation of the genome. As a result, certain types of genetic variations, such as silent substitutions within codons or changes in coding regions of genes, may not be detectable through protein electrophoresis [19]. To overcome these limitations and achieve a more comprehensive understanding of genetic variations, other molecular techniques, such as DNA-based methods like PCR-RFLP or sequencing, are often employed. These methods allow for the direct analysis of the DNA sequence and provide more detailed information about genetic variations at specific loci.

Milk protein variants have been employed in various studies for the purpose of characterizing different cattle breeds [20] and investigating the evolutionary aspects of both animal resources and milk protein genes [21]. 

β-lactoglobulin (β-Lg) polymorphism in cattle is extensively studied, providing a wealth of detailed information. A genome-wide association study for content in bovine milk revealed the presence of three major regions that significantly influence the composition of milk protein, in addition to several regions with smaller effects involved in the regulation of milk protein composition using a 50K single nucleotide polymorphism (SNP) chip [22].

The existence of hemoglobin polymorphism in Algerian hill cattle was initially documented in a study [23], and subsequent research has focused on various aspects of bovine milk in Algeria. These investigations have explored physicochemical characteristics [24], the control of milk's sanitary quality [25], and microbiological quality [26]. However, no specific studies on β-lactoglobulin (β-Lg) polymorphism in Algerian local cattle have been conducted thus far. A genetic characterization of three local bovine populations was carried out by SNP markers on the Illumina BovineSNP50 chip [27].

The advent of the Polymerase Chain Reaction - Restriction Fragment Length Polymorphism (PCR-RFLP) technique has revolutionized the analysis of genetic polymorphisms, allowing for the investigation of variations in numerous genes, including those encoding β-lactoglobulin [28]. DNA restriction enzymes are enzymes that recognize specific DNA sequences, known as recognition sites, and catalyze the cleavage of DNA at or near these sites. These enzymes are classified as endonucleases because they cleave the DNA molecule internally. An electric field is then applied, causing the DNA fragments to migrate through the gel matrix. The smaller DNA fragments move more quickly, while larger fragments move more slowly [29].

The aim of this study was to genotype a SNP responsible for the amino acid changes in the β-lactoglobulin (β-Lg) variants A and B. The genotyping was performed using the PCR-RFLP method. Additionally, the study had to determine the allelic and genotype frequencies in three indigenous cattle populations in Algeria, namely Chelifienne, Cheurfa, and Guelmoise. Notably, this analyze is the first of its kind conducted in Algeria, providing valuable insights into the genetic variations of β-lactoglobulin in Algerian cattle populations.

## MATERIAL AND METHODS

**DNA sampling: **For this study, a total of 85 unrelated DNA samples were utilized, representing three local Algerian bovine populations. The Chelifienne population consisted of 28 individuals, the Cheurfa population had 31 individuals, and the Guelmoise population included 26 individuals. These DNA samples were collected from the respective populations and were made available for analysis in the study [27]. The use of a sufficient number of samples from each population allows for robust genetic characterization and assessment of the β-lactoglobulin gene polymorphism in Algerian cattle.

**PCR-RFLP analysis: **PCR amplified fragment enclosed 89 bases of Exon IV and 158 bases of Intron IV of the β-lactoglobulin gene. The PCR was carried out using a Stratagene thermal cycler. The reaction mixture was composed of (200 ng) genomic DNA, 1 X PCR buffer, 1.5 mM MgCl_2_, 0.2 mM dNTPs, 0.1 µM of each primer and 0.5 U* Taq* DNA Polymerase in a total volume of 25 µl. Primers used were according to Medrano and Aguilar-Cordova [17]; Forward JBLG2: 5'TGTGCTGGACACCGACTACAAAAG-3' and reverse JBLG3: 5'GCTCCCGGTATATGACCACCCTCT-3'. The PCR reactions were carried out in 0.2 ml PCR plates with the following PCR conditions: 1 cycle of initial denaturation for 5 minutes at 94°C, 30 cycles of 30 seconds at 94°C, 58 minutes at annealing temperature, 30 seconds at 72°C, and 1 cycle of final extension for 10 minutes at 72°C.

Following amplification, the resulting DNA fragments underwent analysis using electrophoresis on a 2% agarose gel. To determine the size of the amplified fragments, a commercial 50 bp ladder was used as molecular weight marker (Takara, Bio Europe)*. *Then the amplified DNA fragments were visualized under UV light by edithium bromure coloration. The sizes of each allele were estimated in accordance with the DNA ladder size standards. The PCR products were subjected to digestion by restriction enzymes in a total volume of 30 µl. The reaction was set up with 10 µl of PCR product, 1 X buffer for restriction enzyme (SibEnzyme, Russia), 0.5 units of the restriction enzyme *Hae*III (10U/µl) (Takara, Bio Europe) and incubated at 37°C for 16 h according to [17].

The restriction fragments obtained from the digestion of the PCR products were separated and analyzed by electrophoresis on a 2% agarose gel. The length of the fragments was analyzed with a 50 bp ladder. This analysis allows for the determination of the fragment lengths generated by the restriction enzyme digestion, providing information about the presence or absence of specific restriction sites and the genotypes of the β-lactoglobulin gene variants in the samples.

**Statistical analysis: **The genotype and allele frequencies of the β-Lg locus were estimated by direct counting. Allele frequencies in a sample were estimated from counts of alleles in DNA genotypes. In the context of population genetics and the Hardy-Weinberg theorem, the number of homozygotes and heterozygotes in a sample can be scored and observed genotype frequencies computed. Considering the two-allele case, where *p* is the population frequency of allele *A* (writing *p = f(A)* and *q* is the frequency of allele* B* *(q = f(B),* the Hardy Weinberg theorem states that expected genotype frequencies are *f(AA)* *= p*^2^, *f(AB) = 2pq* and *f(BB) = q*^2^. The Pearson’s chi-square (χ^2^) (p-value<0.05) was used to check whether the population is in Hardy-Weinberg Equilibrium (HWE) or not. Genotype frequencies are additional basic genetic parameters of a population. 

## RESULTS AND DISCUSSION

**Figure 1:**
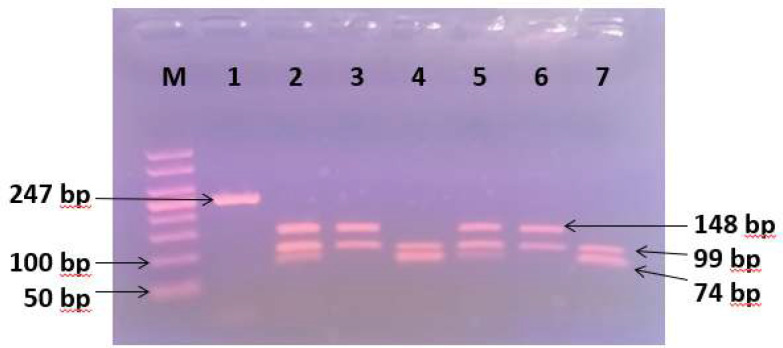
Agarose gel separation of b-lactoglobulin alleles from PCR-amplified genomic DNA Ethidium bromide stained gel (2% agarose). Lane 1: 50 bp marker Lanes 2: amplified 247 bp segment corresponding to DNA b-lactoglobulin PCR; Lane 3 and 6 genotypes β-Lg^A/A^, Lane 2 and 5 genotype β-Lg^A/B^ Lanes 4 and 7 genotype β-Lg^B/B^

In this study, the β-Lg gene locus amplification in the three cattle populations was initiated using the JBLG2 and JBLG3 primers, resulting in a PCR product of 247 base pairs (bp). The subsequent *Hae*III analysis allowed for the identification of three genotypes: β-Lg^A/A^, β-Lg^B/B^, and β-Lg^A/B ^(Fig. 1). The sizes of the fragments corresponding to each genotype are presented in Table 1.

**Table 1:** Genotypic freqeucnies of β-lactoglobulin in Algerian local cattle populations

**Genotypes**	**Fragment size after digestion with ** ***Hae*** **III restriction enzyme**	**Local cattle populations**		
PCR-Product (bp)	247 bp	Chelifienne (*n *= 28)	Cheurfa (*n*= 31)	Guelmoise (*n* = 26)
β-Lg^A/A^	148 bp and 99 bp	02	02	13
β -Lg^A/B^	148 bp, 99 bp and 74 bp	12	14	13
β -Lg^B/B^	99 bp and 74 bp	14	15	00

n: number of samples

In this study, the β-Lg^B/B^ genotype frequencies were found to be the highest in the three cattle populations studied. The β-Lg^A/B ^genotypes were more frequent in both Chelifienne and Cheurfa cattle, while the A allele frequency is not too high in the populations studied and in close agreement with the results of workers in Najdi and Buffalo cattle, the B allele (0. 91 and 0.81) were higher than that of A allele (0.08 and 0.18) [30] and frequencies of A and B alleles were 0.17 and 0.83 and 0.39 and 0.61 in Sahiwal and Tharparkar breeds, respectively [31]. The genotypic frequencies of β -Lg^A/A^, β -Lg^A/B^ and β -Lg^B/B^ were 0.08, 0.41, 0.50 in Chelifienne; 0.08, 0.41, 0.50 in Cheurfa and 0.01, 0.19, 0.56 in Guelmoise, while the frequency of AA genotype was found to be absent in Guelmoise population and very low in both Chelienne and Cheurfa populations. The gene frequencies of the A and B alleles were 0.29 and 0.71 in Chelifienne and Cheurfa and 0.25 and 0.75 in Guelmoise, respectively. The results of the present study provide further confirmation that *Bos taurus* cattle have predominantly exhibit the β-Lactoglobulin B type. The genotype frequencies of β -Lg^B/B^ and β -Lg^A/B^ were found to be higher than compared to the β -Lg^A/A^ genotype in all the animals studied. 

It is worth noting that no previous studies have investigated the association between beta-lactoglobulin genotype and milk proteins in Algerian cattle. According to [32], the level of β -lactoglobulin in milk was found to be significantly higher in homozygous β -Lg^A/A^ than in homozygous β -Lg^B/B^. There is a relationship between  -lactoglobulin polymorphism on the one hand, casein level and casein number on the other hand ( -Lg^B/B^> β -Lg^A/B^> β -Lg^A/A^). The higher casein number is higher in β -Lg^B/B^ cow's milk, which tends to increase the cheese yield as much, through a curd of better consistency [4].

The information you provided suggests that studies have shown associations between β-lactoglobulin polymorphism and milk composition, specifically in relation to whey protein, casein protein, fat, and total solids concentrations. Other studies have revealed that bulk milk with the β-lactoglobulin AA phenotype exhibits a 28% increase in whey protein concentrations compared to β-lactoglobulin BB phenotype bulk milk. Additionally, the β-lactoglobulin AA phenotype bulk milk demonstrates a 7% decrease in casein protein concentrations, an 11% decrease in fat concentrations, and a 6% decrease in total solids concentrations. The elevated whey protein concentrations observed in β-lactoglobulin AA phenotype bulk milk are attributed to significant enhancements in β-lactoglobulin levels within this type of milk [33].

The milk of the animal with the β -Lg^B/B^ genotype yielded significantly more cheese than the milk of animals that carry the β -Lg^A/A^ genotype in Indian cross-bred (*Bos taurus* × *Bos indicus*) dairy bulls [34]. Allele β -Lg A was associated with increased β -Lg concentration and percentage of β -Lg to total whey protein and with decreased content of other milk proteins [35].

On the other hand, the genotype β -Lg^A/B^ was also found to be associated with protein content in Holstein cows [36] and that genotypes  -Lg^A/A^ and β -Lg^B/B^ had significantly (p<0.05) higher total milk yield and peak yield compared to β -Lg^B/B^ [37].

The study of first-calf cows of the Kholmogorskaya breed of the Tatarstan type and black-motley × Holstein cows has shown that the first-calf cows with the genotype β -Lg^B/B^ and β -Lg^A/B^ had the best cheese-making properties of milk. These animals were superior to their coevals with the β -Lg^A/A^ genotype in terms of the highest yield of the desired dense casein clot and the shortest duration of milk clotting time [38].

### Acknowledgement:

This work was supported by a Laboratory of Cellular Molecular Genetics, the University of Sciences of Technology “Mohamed Boudiaf”, Oran, Algeria.

### Conflict of Interest:

Authors must indicate whether or not they have a financial relationship with the organization that sponsored the research. 

### Authors’ contribution:

NB was involved in study conception, data collection, data analysis, interpretation and anucript preparation. NTA contributed to manuscript correction. All authors reviewed, approved the final manuscript and submitted to publication. 
